# The role of macropinocytosis in the propagation of protein aggregation associated with neurodegenerative diseases

**DOI:** 10.3389/fphys.2015.00277

**Published:** 2015-10-16

**Authors:** Rafaa Zeineddine, Justin J. Yerbury

**Affiliations:** ^1^Illawarra Health and Medical Research Institute, University of WollongongWollongong, NSW, Australia; ^2^Faculty of Science, Medicine and Health, School of Biological Sciences, University of WollongongWollongong, NSW, Australia

**Keywords:** protein aggregation, amyloid fibrils, macropinocytosis, endocytosis, propagation

## Abstract

With the onset of the rapidly aging population, the impact of age related neurodegenerative diseases is becoming a predominant health and economic concern. Neurodegenerative diseases such as Alzheimer's disease, Creutzfeldt-Jakob disease (CJD), Parkinson's disease, Huntington's disease, frontotemporal dementia (FTD), and amyotrophic lateral sclerosis (ALS) result from the loss of a specific subsets of neurons, which is closely associated with accumulation and deposition of specific protein aggregates. Protein aggregation, or fibril formation, is a well-studied phenomenon that occurs in a nucleation-dependent growth reaction. Recently, there has been a swell of literature implicating protein aggregation and its ability to propagate cell-to-cell in the rapid progression of these diseases. In order for protein aggregation to be kindled in recipient cells it is a requisite that aggregates must be able to be released from one cell and then taken up by others. In this article we will explore the relationship between protein aggregates, their propagation and the role of macropinocytosis in their uptake. We highlight the ability of neurons to undergo stimulated macropinocytosis and identify potential therapeutic targets.

## Introduction

Neurodegenerative diseases are closely linked to the formation and deposition of protein aggregates, quite often fibrillar, that accumulate intracellularly, such as α-synuclein in Parkinson's disease (PD), or extracellularly, such as the amyloid-beta (Aβ) peptide plaques associated with Alzheimer's disease (AD) (Chiti and Dobson, 2006). Although the peptides and proteins that aggregate are seemingly unrelated in terms of primary or tertiary structure, the resulting deposits are remarkably similar, often sharing a rope-like fibrillar morphology, a common cross-β core structure, and the ability to bind specific dyes such as thioflavin T and Congo red (Dobson, 2003). It has been postulated that oligomeric species present in solution prior to the appearance of fibrils are more likely to be responsible for cellular toxicity (Kayed et al., 2003). However, it is probable that all aggregate species provoke some level of cellular stress, although how protein aggregates induce cell injury is not fully understood. One potential mechanism of toxicity is mediated through exposed hydrophobic residues found on protein aggregates that have been shown to interact with cell surface receptors and membranes (Stefani and Dobson, 2003; Bolognesi et al., 2010), leading to membrane disruption and inappropriate signaling cascades.

**Figure 1:**
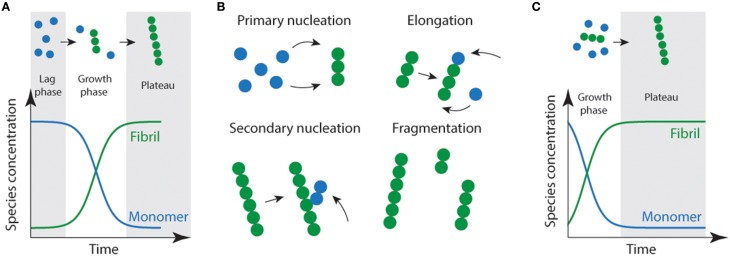
**Schematic representation of amyloid fibril formation. (A)** Fibril formation can be characterized by a lag phase where nucleation events occur, following critical nucleation a growth/elongation phase is observed which can proceed via primary (monomer addition) or secondary (fragmentation/secondary nucleation) events **(B)**. During the latter stages, mature fibrils are formed which often display strong ThT emission signals. **(C)** Addition of fibrils or other functional seeds to the start of the reaction allows elongation to proceed without the requirement for primary nucleation removing the lag phase.

Protein aggregation or fibril formation can be described as a nucleated self-assembly reaction. In this context, misfolded monomeric proteins or peptides must first aggregate to form stable nuclei from which fibril growth can occur via addition of further monomers. *In vitro*, using bulk measurements such as light scattering or thioflavin T fluorescence, protein aggregation reactions display sigmoidal growth kinetics (Figure 1). Initially, there is a lag phase which is thought to reflect the time it takes for the nuclei to form, and where the formation of fibrils is below the threshold of detection (Serio et al., 2000; Pedersen et al., 2004). In solution, there are two predominant species; monomers and fibrils (Figure 1A). While oligomeric aggregates are thought to be present in small amounts (i.e., < 2% of total species). During the elongation phase, the concentration of fibrils increases dramatically as the monomer concentration decreases. This is thought to be due to increasing numbers of actively growing fibrils via fragmentation of fibrils creating new “growing ends” and/or secondary nucleation (Figure 1B; where available sites on existing fibrils catalyze the nucleation of new aggregates) (Chiti and Dobson, 2006; Wilson et al., 2008; Arosio et al., 2014; Knowles et al., 2014). The lag phase can be circumvented by the addition of exogenous nuclei or “seeds” in the form of preformed fibrils (Jarrett and Lansbury, 1993). As a consequence of seeding, the lag time is eliminated (Jarrett et al., 1993) resulting in a first-order growth polymerization (Figure 1C).

## Patterns of neurodegenerative pathology in humans

In major neurodegenerative diseases, such as Amyotrophic Lateral Sclerosis (ALS), Frontotemporal Dementia (FTD), AD, PD, and Huntington's diseases (HD), pathological changes such as loss of neurons and presence of pathological protein aggregates, typically follow distinctive anatomical patterns. The observed patterns are consistent with a spreading of pathology from one part of the brain to another (Brundin et al., 2010). The progression of these disorders, which is also associated with increasing clinical severity, has enabled the development of several staging systems for a range of neurodegenerative diseases (Braak et al., 2003, 2006; Brettschneider et al., 2013, 2014). The resulting patterns suggest that pathology is not only propagated between nearby cells, but that it also remotely connects regions of the brain along axonal pathways (Brundin et al., 2010; Brettschneider et al., 2015).

## Propagation of protein aggregation and neurodegenerative disease

The patterns identified in neuropathological studies are consistent with propagation of protein misfolding and aggregation reminiscent of the prion diseases (Aguzzi, 2009). Prion diseases include human disorders such as Kuru and Creutzfeldt-Jakob disease (CJD), but also exist in animal populations in the form of scrapie in sheep, and bovine spongiform encephalopathy (BSE) in cattle (and several other species have their own version of prion-opathies). Prion diseases are infectious neurodegenerative diseases where the pathogenic agent is a misfolded/aggregated form of the prion protein which can seed misfolding and aggregation of the normal cellular prion protein in naïve cells, and indeed in naïve hosts transmitting pathology between cells and between individuals (Prusiner, 1998). The neurodegenerative diseases described in this review differ from prion diseases in one crucial respect; they are not infectious. Importantly, most neurodegenerative diseases do not involve the prion protein but typically a disease specific misfolded protein. However, it is useful to model the progression of neurodegenerative diseases associated with protein aggregation on the propagation of misfolded prion protein within an individual host. It may help provide an explanation for the apparent patterns of pathology in ALS, PD, and AD.

It has been known for decades that fibril formation can be seeded *in vitro* through addition of preformed fibrils, removing the rate limiting lag phase (see Figure 1). Aβ from the brains of AD patients can seed amyloid formation in non-human primates (Baker et al., 1993) and in mice engineered to produce large amounts of APP (Kane et al., 2000). This propagation is easily understandable given that Aβ aggregates do not have to cross plasma membranes to seed further aggregation, as amyloid plaques are present outside of cells. One might imagine that it would be less likely that aggregates formed inside one cell could propagate aggregation in another. However, there is a wave of research that demonstrates that this is possible (Clavaguera et al., 2009; Desplats et al., 2009; Ren et al., 2009). Evidence suggests that large fibrillar aggregates of a range of proteins (tau, α-synuclein, polyglutamine repeats, SOD1) are able to gain access to the cytoplasmic compartment via an incompletely understood mechanism and propagate misfolding and aggregation (Clavaguera et al., 2009; Desplats et al., 2009; Ren et al., 2009; Grad et al., 2014). Injection of brain/spinal cord extracts from transgenic mice expressing human tau, SOD1 or α-synuclein can seed pathology in the sites of injection and spread to other regions of the nervous system (Clavaguera et al., 2009; Mougenot et al., 2012; Ayers et al., 2014). Further, cell culture experiments have shown that these neurodegenerative disease associated protein aggregates can propagate cell-to-cell in neuronal and other cell lines, and into primary neurons (Hansen et al., 2011; Münch et al., 2011; Volpicelli-Daley et al., 2011; Furukawa et al., 2013; Guo and Lee, 2013; Grad et al., 2014). Moreover, cell culture experiments also show that insoluble material from brain tissue can seed aggregation of neuropathological TDP-43, whose cytoplasmic accumulation is associated with ALS (Furukawa et al., 2011). It is clear that these disease-associated aggregates are able to spread to naïve cells in culture gaining access to the cytosol in an unknown manner linked with fluid phase endocytosis. It is vital then to understand this mechanism to identify targets that will halt passage of the “infectious” particle in a strategy analogous to drugs blocking viral entry. Logically there are two vital steps that must occur in order to propagate aggregation between cells; aggregates must first be released in to the extracellular space from the cells in which they are made, and then be taken up by nearby cells to seed further aggregation in the cytosol of naïve cells.

## Aggregates can be released by neurons

**Figure 2:**
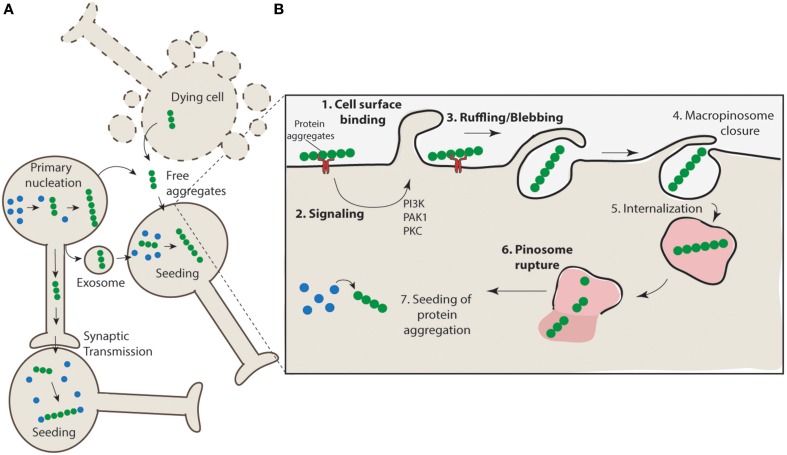
**Propagation of aggregation and a proposed mechanism for aggregate uptake via macropinocytosis. (A)** Protein aggregates form in neurons (primary nucleation) and have the potential to further nucleate the aggregation of other proteins. These protein aggregates can transfer directly from cell-to-cell through synaptic transfer, or be actively released via secretion mechanisms (e.g., exosomes) or in their naked form, either actively released from live cells or non-specifically released from dying cells, to neighboring interconnecting neurons. The uptake of such aggregates nucleates aggregation in naïve cells. **(B)** Aggregates may interact with cell surface receptors (such as HSG) (1) and promote the clustering and activation of signaling receptors such as receptor tyrosine kinases. This may result in the activation of signaling pathways such as those regulated by PAK1 and PKC (2) and subsequent mobilization of actin and formation of ruffles/blebs (3). Upon macropinosome closure (4) the structure is internalized (5). Given the unstructured nature of the macropinosomes it is likely that rigid aggregate structures may cause rupture (6) and allow access of the aggregates to the cytosol where nucleation of aggregation can proceed (7).

The mechanism of release of protein aggregates is incompletely understood, and it has been proposed that both active and passive routes of escape may be responsible for aggregate release (Brundin et al., 2010). For example, cell death may promote the non-specific release of aggregates from the cell or there may be active mechanisms responsible for release through exocytosis pathways (Figure 2A). SOD1 aggregates are generally found to be intracellular in human SOD1-linked familial ALS patient samples, however, there is evidence from cell culture models that these aggregates can escape from neurons and come into contact with nearby cells (Münch et al., 2011; Grad et al., 2014). There is evidence of α-synuclein release from neurons into the extracellular space via brefeldin A-insensitive unconventional exocytosis (Lee et al., 2005; Jang et al., 2010). The released α-synuclein is thought to be misfolded or aggregated (Jang et al., 2010) and is consistent with β-sheet-rich α-synuclein oligomeric aggregates (Kim et al., 2013). Tau aggregates have also been shown to be able to transfer between cells (Frost et al., 2009; Frost and Diamond, 2010; Dujardin et al., 2014). As in the case of anti-SOD1 antibodies (Grad et al., 2014) the use of anti-tau antibodies can block this tau transfer (Yanamandra et al., 2013). While cell lysates from mutant huntingtin-expressing cells promote propagation of aggregation in naïve acceptor cells, huntingtin aggregate release from neurons has not been conclusively demonstrated (Ren et al., 2009). The mechanism underpinning misfolded SOD1 release in cell culture models has been shown to be linked to both the release of free aggregates associated with cell death and to active release via exosomes (Grad et al., 2014). Collectively these data leave open the possibility that both cell death and active secretion mechanisms are acting in concert to promote aggregate release *in vivo*.

## Aggregates can be taken up by neurons

In order to facilitate the propagation of intracellular aggregation in neurodegenerative diseases, such as ALS, PD, AD, and HD, active aggregate nuclei or seeds must gain access to the cytosol of naïve cells. Active nuclei could be large aggregates such as those that are macroscopically visible and accumulating in neurons (>2 microns in size), or small soluble oligomeric aggregates that might diffuse between cells. Fibrillar aggregates are generally 1–20 nm across and can be hundreds of nm long (Dobson, 2003), large aggregates that accumulate many fibrils in cells can be several μm in diameter (Farrawell et al., 2015). In this light, and given that neurons are not professional phagocytes it would be reasonable to assume that endocytosis of protein aggregates by neurons would be almost impossible. However, previous work has shown that exogenously applied SOD1 aggregates associated with ALS can be taken up efficiently and rapidly into living cells (Furukawa et al., 2013), such as neuronal cells (Neuro2a; N2a, NSC-34, and SH-SY5Y), in a time dependent manner (Münch et al., 2011; Sundaramoorthy et al., 2013).

In the context of PD, direct cell-to-cell transfer of aggregated α-synuclein has been demonstrated in humans, cell culture and animal models (Desplats et al., 2009; Lee et al., 2010). Post mortem studies were able to show that approximately 2–5% of normal embryonic neurons transplanted in the brains of PD patients acquired α-synuclein rich Lewy bodies over a period of 5 years (Brundin et al., 2008; Hansen et al., 2011). This phenomenon was also demonstrated in mice, where α-synuclein was shown to propagate from mouse host brain cells to grafted dopaminergic neurons (Hansen et al., 2011). Cell culture studies have shown that extracellular α-synuclein in various forms (fibrils, oligomers, and monomers) can be internalized by cultured neuronal cells (Lee et al., 2008). Furthermore, mouse cortical neuronal stem cells were shown to internalize extracellular aggregated α-synuclein either applied as recombinantly produced protein aggregates or from co-culture with cells overexpressing and subsequently releasing α-synuclein aggregates (Desplats et al., 2009). Together, these findings provide evidence that neurons are capable of engulfing even large α-synuclein structures. Tau aggregates, associated with AD and tauopathies such as FTD, have also been shown to be taken up into neurons. A recent study has shown that recombinant synthetic tau fibrils have been internalized in primary neurons derived from embryonic mouse hippocampus, and this triggered robust aggregation of endogenous soluble protein (Guo and Lee, 2013). Similarly, cell culture studies have shown that fibrillar polyQ aggregates (K_2_Q_44_K_2_), a model for polyQ expansion in huntingtin associated with HD, can efficiently enter into N2A cells and gain access to the cytosol to potentially nucleate the aggregation of otherwise soluble proteins (Ren et al., 2009). Collectively these data show that, despite their size, aggregates from a range of neurodegenerative diseases can be efficiently taken up by cells and most importantly this can occur in neurons. Given the similar structure and large size of these aggregates it is likely that similar mechanisms are used by subsets of neurons to engulf such large aggregates. Intuitively, the large size of the protein aggregates argues against neuronal entry by caveolae (generally used for particle sizes from 50 to 100 nm) (Richter et al., 2008) or clathrin-coated pits (for particle sizes < 200 nm; Traub, 2009). One potential mechanism that may explain the uptake of such large structures is the process of macropinocytosis.

## Hijacking macropinocytosis for aggregate entry into cells

Macropinocytosis is a transient, actin-dependent process that leads to the internalization of fluid, membrane and other particles into large vacuoles (up to 5 μm). It is generally triggered by growth factors but can be triggered by a variety of particles such as bacteria, apoptotic bodies, necrotic cells and viruses (Mercer and Helenius, 2012). The activation of macropinocytosis induces membrane ruffling (or membrane extensions or even blebbing) that can fold back on to the cell, fusing with the plasma membrane and forming large fluid filled randomly sized vacuoles that lack supporting coating molecules (Meier et al., 2002). Several studies now suggest that macropinocytosis may be involved in the uptake of protein aggregates associated with various neurodegenerative diseases (Münch et al., 2011; Holmes et al., 2013; Sundaramoorthy et al., 2013; Grad et al., 2014; Tang et al., 2015).

Studies on the propagation of SOD1 aggregation by Münch et al. were the first to suggest that mutant SOD1 ALS protein aggregates enter into N2A cells *via* macropinocytosis (Münch et al., 2011). The work used a large panel of inhibitors of a range of cellular functions to systematically rule out specific pathways of endocytosis such as caveolin and clatherin-dependent endocytosis (Münch et al., 2011). Subsequently, these results were confirmed by a study that showed that uptake of both extracellular wild-type and mutant SOD1 soluble forms into NSC-34 cells can be inhibited by small molecule inhibitors of macropinocytosis EIPA and rottlerin (Sundaramoorthy et al., 2013). Similarly, EIPA was shown to inhibit the uptake of human wild-type SOD1 aggregates into NSC34 cells (Grad et al., 2014) consistent with macropinocytosis. EIPA (ethylisopropylamiloride) is an analog of amiloride that is an inhibitor of Na^+^/H^+^ exchangers. It is thought to be specific to macropinocytosis due to the susceptibility of GTPases such as Rac1 to pH changes (Koivusalo et al., 2010).

More broadly, uptake and cell-to-cell transmission of α-synuclein into neurons has been shown to be mediated *via* an unconventional endocytosis pathway (Lee et al., 2008; Desplats et al., 2009). Also, while initially synthetic polyglutamine fibrils were proposed to enter cells via direct penetration of the lipid bilayer (Ren et al., 2009) cell surfaces structures have been recently implicated in the binding and internalization of both synthetic polyglutamine (K_2_Q_44_K_2_) and huntingtin exon 1 (Htt exon_1_Q44_44_) fibrils suggesting a role for endocytosis mediated uptake (Trevino et al., 2012). A recent study was also able to show that the amyloid precursor protein (APP) can be rapidly internalized from the cell surface to lysosomes, bypassing early and late endosomes *via* macropinosome-like structures. This process was found to be mediated by Arf6, a regulator of macropinocytosis. A dominant negative mutant version of Arf6 inhibits transport of APP to lysosomes, and therefore reduces the secretion of Aβ (Tang et al., 2015). Further, exogenously added AD associated-recombinant tau fibrils have also been shown to be taken up by cultured cells in a process consistent with pinocytosis suggested by the co-localization of tau aggregates with Dextran, a marker of fluid phase endocytosis (Frost et al., 2009). In support of these findings, a recent study was able to show that small misfolded tau species are also internalized through the process of bulk endocytosis (Wu et al., 2013). Further to this, uptake of fibrillar tau into C17.2 cells (mouse neural stem cells) could be inhibited by both amiloride and rottlerin consistent with macropinocytosis (Holmes et al., 2013). The same study demonstrates that tau fibrils could be observed in vacuoles and invaginations with diameters of approximately 5 μm. Lastly, in a result that suggests stimulated macropinocytosis has been activated, the uptake of the fluid phase marker dextran was increased with increased doses of tau fibrils (Holmes et al., 2013). This uptake could be inhibited by suppressing cell binding of tau fibrils by blocking binding to heparan sulfate proteoglycans, indeed this also blocked uptake when aggregates were injected in to the brains of mice (Holmes et al., 2013). This was also shown in a more recent study where recombinant Tau assemblies (trimers) bound heparan sulfate proteoglycans on the cell surface to mediated Tau uptake and seeding into primary cortical neurons and HEK293 cells (Mirbaha et al., 2015). This binding stimulated macropinocytosis. However, how the binding to heparan sulfate proteoglycans on the cell surface relate to activation of macropinocytosis remains unclear.

In the context of cell-to-cell transfer of misfolded or aggregated protein, it is interesting to note that exosomes can contain misfolded and aggregated protein (Bellingham et al., 2012), and further, that exosomes can enter cells via macropinocytosis (Fitzner et al., 2011). However, additional work needs to be performed to confirm a role of macropinocytosis in exosome associated protein misfolded propagation.

## Macropinocytosis in neurons

The fact that neurons can engulf large particles such as protein aggregates at all seems counterintuitive. Endocytosis of large particles is usually thought of as restricted to professional phagocytes such as macrophages and microglia (Swanson, 2008; Mercer and Helenius, 2012). This is useful for engulfment of viral particles, bacteria, and fragments of dying cells in order to dispose of them. Without extensive degradation machinery, such as in macrophages, neurons are seemingly left vulnerable with the ability to take up large particles whilst not built to remove them. So why is it that neurons have the capacity to perform such a function? It is unlikely that neurons have evolved to specifically endocytose pathogens and large protein aggregates. Although, several viruses are known to enter neurons via macropinocytosis (Talekar et al., 2011; Hollidge et al., 2012; Kalia et al., 2013), this is likely due to the hijacking of machinery involved in macropinocytosis evolved for other purposes. Macropinocytosis, or closely related processes, are thought to regulate growth cone membrane recycling and are an integral part of growth cone collapse, axon retraction and turning during development and injury (Jurney et al., 2002; Tom et al., 2004; Kolpak et al., 2009; Kabayama et al., 2011). The membrane recycling process in growth cones is associated with actin-dependent membrane ruffles that fuse back on the plasma membrane creating large pinosomes dependent on PI3K and Rac1; all characteristic of macropinocytosis. Upon synaptogenesis this process is suppressed, consistent with the idea that mature neurons do not undergo bulk changes in the membrane via macropinocytosis (Bonanomi et al., 2008). However, axonal injury in *in vitro* and *in vivo* models triggers axonal remodeling in adult neurons that is associated with large amounts of fluid uptake (Tom et al., 2004) consistent with macropinocytosis. Interestingly, some disease associated mutations such as those in ALS2 (in ALS) and γPKC (in spinocerebellar ataxia 14) result in dysregulation of macropinocytosis (Otomo et al., 2008; Yamamoto et al., 2014) suggesting dysfunction of these processes are detrimental to large neurons.

## Therapeutic targeting of macropinocytosis for neurodegenerative diseases

Collectively, the data summarized above suggests that macropinocytosis plays a role in allowing the passage of protein aggregates into naïve cells. Understanding neuron specific macropinocytosis mechanisms will be vital in identifying a target to slow disease progression. In particular, the intracellular pathways that result in activation of macropinocytosis, the formation and closure of macropinosomes, and importantly potential disintegration of macropinosomes must be examined. In an analogous situation, mechanisms underpinning Ebola virus entry *via* macropinocytosis are being scrutinized, with promising compounds targeting endocytosis and escape of viral particles from endosomes proving successful in mice (Sakurai et al., 2015). Macropinocytosis may be a viable target given that other cell types such as microglia that clear protein aggregates appear to be *via* different pathways (Roberts et al., 2013). It may be possible then to redirect aggregates from entering neurons by suppressing macropinocytosis in pathological conditions while maintaining receptor mediated phagocytic pathways utilized by microglia to engulf protein aggregates.

One potential target could be the cell surface receptor triggering activation of macropinocytosis (Figure 2B). Recent work suggests heparan sulfate proteoglycans are involved in the entry of tau aggregates, but how this relates to activation of macropinocytosis and to entry of other neurodegenerative disease associated aggregates is unclear (Holmes et al., 2013). Although there is no evidence in the context of aggregate activated macropinocytosis, previous work would suggest that receptor tyrosine kinases are involved in endocytosis via macropinocytosis (Kerr and Teasdale, 2009). Activation of receptor tyrosine kinases causes an increase in actin polymerization at the cell surface, resulting in an elevation in actin-mediated ruffling and therefore an increase in macropinosome formation, which is the mechanism distinguishing it from other endocytic pathways (Kerr and Teasdale, 2009). In addition to this, inhibition of RAC1 activity inhibits ruffle formation (Figure 2B) irrespective of receptor tyrosine kinase signaling (Lanzetti et al., 2004). These are two examples of pathways that could potentially be exploited to slow or stop the progression of toxic protein aggregates that enter cells *via* macropinocytosis.

## Conclusions

Propagation of protein aggregation is implicated in the progressive nature of several neurodegenerative diseases such as AD, PD, and ALS. This may be explained by the nucleation of aggregation in nearby or connected neurons. In the cases where protein aggregates are intracellular, in order for this propagation to occur aggregates must be able to gain access to the cytosol of naïve neurons. Evidence is accumulating that implicates neuronal macropinocytosis in the uptake of protein aggregates. This may provide rational therapeutic targets that could stop the spread of pathology and halt these progressive neurodegenerative diseases in their tracks.

## Funding

JJY is supported by a NHMRC career development fellowship 1084144.

### Conflict of interest statement

The authors declare that the research was conducted in the absence of any commercial or financial relationships that could be construed as a potential conflict of interest.
